# An Overview of Various Additive Manufacturing Technologies and Materials for Electrochemical Energy Conversion Applications

**DOI:** 10.1021/acsomega.2c05096

**Published:** 2022-11-03

**Authors:** Bulut Hüner, Murat Kıstı, Süleyman Uysal, İlayda Nur Uzgören, Emre Özdoğan, Yakup Ogün Süzen, Nesrin Demir, Mehmet Fatih Kaya

**Affiliations:** † Engineering Faculty, Energy Systems Engineering Department, Heat Engineering Division, Erciyes University, 38039 Kayseri, Turkey; ‡ Engineering Faculty, Department of Mechanical Engineering, Erciyes University, 38039 Kayseri, Turkey; § Erciyes University H2FC Hydrogen Energy Research Group, 38039 Kayseri, Turkey; ⊥ BATARYASAN Enerji ve San. Tic. Ltd. Şti, Yıldırım Beyazıt Mah., Aşık Veysel Bul., ERÜ TGB İdare ve Kuluçka 4, No: 67/3/11, Melikgazi, 38039 Kayseri, Turkey

## Abstract

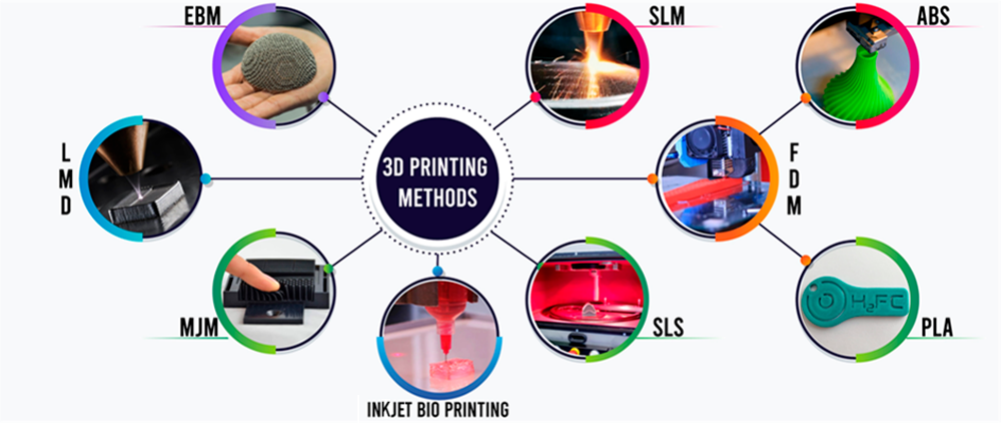

Additive manufacturing (AM) technologies have many advantages, such as design flexibility, minimal waste, manufacturing of very complex structures, cheaper production, and rapid prototyping. This technology is widely used in many fields, including health, energy, art, design, aircraft, and automotive sectors. In the manufacturing process of 3D printed products, it is possible to produce different objects with distinctive filament and powder materials using various production technologies. AM covers several 3D printing techniques such as fused deposition modeling (FDM), inkjet printing, selective laser melting (SLM), and stereolithography (SLA). The present review provides an extensive overview of the recent progress in 3D printing methods for electrochemical fields. A detailed review of polymeric and metallic 3D printing materials and their corresponding printing methods for electrodes is also presented. Finally, this paper comprehensively discusses the main benefits and the drawbacks of electrode production from AM methods for energy conversion systems.

## 1 Introduction

Increasing population growth and rapid industrialization require new research studies to meet these energy demands.^1^ Due to the people’s high growing energy needs, clean and environmentally friendly renewable energy technologies may provide a sustainable solution. To reduce greenhouse gas emissions, many researchers have turned their search to clean and environmentally friendly renewable energy sources. The interest in use of solar energy,^2^ wind energy,^3^ and energy from biomass^4^ applications is increasing day by day. The development of renewable energy systems will be promising for the solution of the most significant tasks, like improving the energy supply security, biofuel economy, solving local energy and water supply problems, and raising the living standard and employment level of the local population.^5,6^ However, high cost is one of the biggest obstacles for the common use of these systems. The problem of access to raw materials, which is among the reasons for high cost, may provide a long-term solution for sustainable development in renewable energy technologies. The widespread use of new technologies, such as AM, may contribute to reduce the carbon footprint. The AM method, which is claimed to be a green technology, has great potential to increase material efficiency, reduce life cycle impact, and reduce the need for special tools in the manufacture of parts. It also provides faster production and more time savings compared to traditional methods. Therefore, the energy consumption in required time and cost to produce small volume parts may be decreased significantly.^7−9^ When the Industrial Revolution is considered, an improvement is expected in the manufacturing process of products. For this reason, three-dimensional (3D) printing technology, known as the AM method, accounts for the basis of the Industrial Revolution (4.0) among new production techniques. AM technology provides the rapid production of parts by adding objects layer-by-layer from computer-aided 3D geometry models without the constraints of traditional machining, forging, and casting processes. Among the rapid production methods, this technology has recently paved the way for the improvement of designs for industrial applications and the rapid production of components. A variety of AM methods and the materials are given in Table 1.

**Table 1:** Various AM Techniques and Their Materials

process	materials	methods	ref
directed energy deposition	metals	laser metal deposition (LMD)	(10−12)
material extrusion	thermoplastic polymers	fused deposition modeling (FDM)	(13−15)
powder bed fusion	plastics, metals and polymers, ceramic powders	electron beam melting (EBM), selective laser melting (SLM)/selective laser sintering (SLS)	(16−18)
material jetting	polymers	multijet modeling (MJM)	(19, 20)
binder jetting	polymers, metals and foundry sands	powder bed and inkjet head 3D printing (PBIH), plaster-based 3D printing (PP)	(21−23)
inkjet bioprinting	biomaterials and human cells	inkjet bioprinting	(24, 25)
sheet lamination	polymers, metals and ceramics	laminated object manufacturing (LOM), ultrasonic additive manufacturing (UAM)	(26, 27)
vat polymerization (VP)	acrylates, epoxides, photoresins, photocurable materials, polymers and ceramics	photopolymerization, digital light processing (DLP), continuous liquid interface production (CLIP)	(28−31)

AM technologies have a great capacity both to decrease material waste through the production stages of products and to reduce energy consumption because it has been determined that there is a significant decrease of up to 27% in global energy demand with the widespread use of AM technologies.^9^ In recent times, these technologies have been widely used in different energy sectors to enhance their performance and increase energy efficiency in the 3D printing of products. It has been especially accepted as one of the new generation solutions for energy storage, energy conversion, and electrochemical applications. For example, in traditional methods, there is a disadvantage to produce flow channels, such as electrodes and bipolar plates for energy applications by machining methods, in terms of both cost and their geometrical structures. Therefore, the AM method has recently become the lead production system in terms of design freedom, material savings, and easy production of complex structures.^32,33^ It was the first application of the photopolymerization method for the 3D printing method. This method was introduced in the 1980s by Hideo Kodama. He developed the method for creating 3D objects by curing a photocuring polymer under ultraviolet (UV) light. It is known as the stereolithography (SLA) method.^34^ The lamination method can be realized by stacking materials on each other after a layer contour definition is obtained with cutting tools in the 3D printing process. The lamination method, which is known as laminated object fabrication (LOM), was discovered at Helisys, Inc. in the late 1980s. In this method, first a layer of material is loaded onto the table and then the profile is created by cutting with a laser or blade.^35^ After the remaining material is removed, a second layer is loaded on top of the first layer. According to the type of materials, such as paper, metal, or plastic, each layer is obtained by sticking to the previous one using adhesive or welding methods.^36^ Another method is an extrusion-based 3D printing process that produces products by directly depositing material with the help of a nozzle after a series of pretreatments (liquefaction process). This technique is known as fused deposition modeling (FDM), which creates 3D printed objects using polymer materials and was explored by Scott Crump in 1989.^37^ The developing 3D printing technology has provided rapid prototyping, which is critical for micro- and macrostructure design in energy applications because 3D printing represents a new manufacturing technique for the production of energy conversion and storage technologies in the production of functional materials for energy applications. Among other advantages, AM technologies offer the unique ability to increase specific performance per unit mass and volume in the manufacture of energy devices with complex shapes.^38^

In this study, the fabrication of 3D printed products using polymer-based and metal powder-based materials, their electrochemical applications and coatings, and the studies of 3D printed products with different geometries are extensively discussed. A future perspective is presented for the new generation energy conversion applications’ research and development (R&D) studies.

## 2 Materials Used in the 3D Printing Method

### 2.1 Polymer-Based Materials

**Figure 1:**
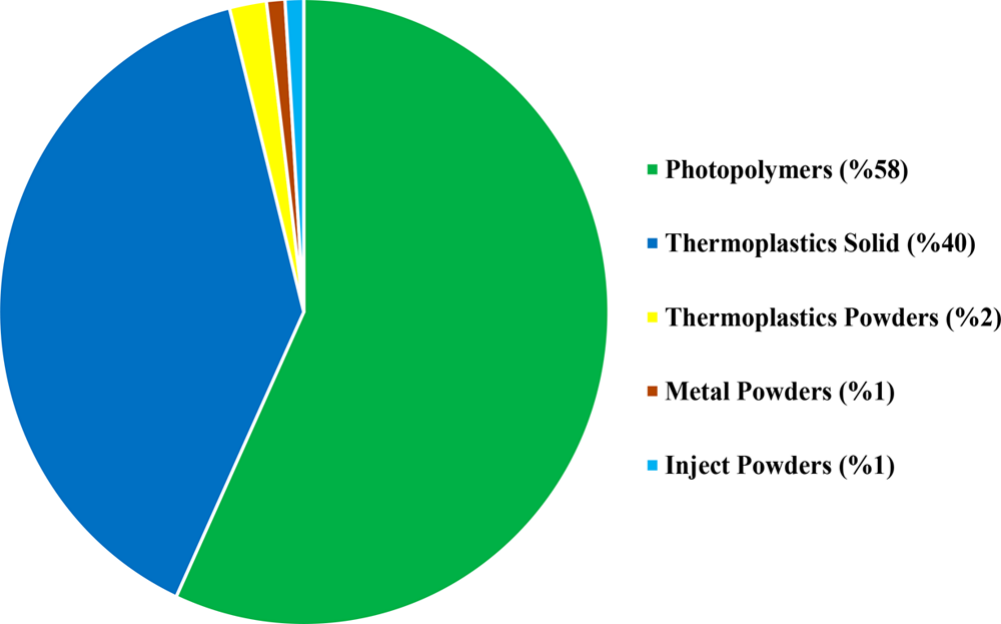
Materials for AM method according to the amount of material consumption by weight in 2014. Reprinted with permission from ref (40). Copyright 2015 Nova Science Publishers.

Polymers are preferred in the AM method due to their easier production and lower cost compared to those of other building materials. In Figure 1, the distribution of consumed polymeric materials for the AM method in 2014 can be seen. Plastic materials represent 99% of the industry, and they are involved in the development of structural mechanical compounds, such as metals.^39^

In the AM method, polymers have the potential to represent many more application than metals in many fields from energy to sustainable applications and health to biomedical. The filaments used in the FDM method represent the largest part of the industry. Although there are several polymeric materials available for AM, they vary in the process of 3D printing depending on the method and their mechanical properties. Polymers, such as acrylonitrile-styrene-butadiene (ABS),^41^ polycarbonate (PC),^42^ polylactic acid (PLA),^43^ polystyrene (PS),^44^ polyamide (PA),^45^ and polyurethane (PU),^46^ are used in the AM method. These materials are used for low-performance components or prototype designs. At the same time, polymers such as polyether ether ketone (PEEK), polyphenylsulfone (PPSU), polyetherimide (PEI), and polyphenylene sulfide (PPS) are used in the AM method due to their heat and chemical resistance.^47−49^ For this reason, the interest in this method is increasing day by day to enhance the mechanical properties of composite and nanocomposite materials or to acquire new functions, like thermal and electrical conductivity for commercially available polymers.^50^ One of the AM methods, “FDM”, is the most preferred method due to its low production costs. With the expiration of Stratasys’ FDM patent after 2009, the spread of FDM machines in the production of 3D printed products was increased. This increase in AM method may accelerate the growth of manufacturing technology of products with the development of new smart materials, nanocomposites, and biomaterials.^37−51^ In energy conversion applications, PLA and ABS-based filaments are the most common thermoplastics.^52^

#### 2.1.1 Polylactic Acid Thermoplastics

PLA is a thermoplastic material that may be obtained from renewable biomass resources, such as starch, corn starch, sugar cane, or tapioca roots, and it belongs to the category of biodegradable polymers.^53^ It has completely biocompostable properties and is able to reduce solid waste disposal problems. PLA-based polymers are preferred mostly in the developing bioplastics industry due to their easy availability and low cost.^54,55^ PLA materials’ mechanical properties, like tensile strength and impact strength, are lower than polypropylene (PPy), poly(ethylene terephthalate) (PET), and poly(ethylene terephthalate) glycol (PETG)-based polymers. Compared to conventional polymers, like PPy, polystyrene (PS), and polyethylene (PE), PLA has a higher mechanical, tensile, and bending strength. As a semicrystalline or amorphous structure, the melting temperature of PLA may change between 55 and 180 °C. The thermal features of PLA can exhibit structural differences according to their molecular weights and compositions.^56^ It can be concluded that PLA has a good stiffness, tensile strength, and gas permeability comparable to those of synthetic polymers, and it is one of the most promising materials to replace petroleum-based polymers in the packaging industry sector. Moreover, in the future research, PLA will be a low-cost material due to their biodegradable properties and simple production of components for industrial applications. Although, nowadays, it has a higher production cost than petroleum-derived plastics, PLA-based polymers may be used in many different practical applications such as agriculture, packaging/food packaging, medical/biomedical industry, energy sector, and automotive industry.

#### 2.1.2 Acrylonitrile-Styrene-Butadiene Thermoplastics

High molecular mass styrene-acrylonitrile copolymers and butadiene-acrylonitrile copolymers were used to fabricate bullet-proof polymer boards during the final years of World War II. These polymers have high impact strength due to their low thermoplastic flow properties. ABS is a product of the systematic polymerization of acrylonitrile, butadiene, and styrene. It has also many properties such as good thermal stability, high resistance, high toughness (even under cold conditions), and hardness. Other important features of ABS polymers are low cost, high strength, and low thermal expansion. Moreover, the development of methods like injection molding and graft polymerization has increased the interest in ABS plastics. ABS also may be uses in many fields like design, fashion, toys, and modern art.^57,58^ The widespread use of FDM techniques has been increased with the utilization of ABS polymers in 3D printers.^59^ In comparison to PLA filaments, ABS polymer filaments require a higher nozzle and bed temperature. They require a wide range of bed temperatures (between 80 and 110 °C) and nozzle temperatures (between 210 and 250 °C) depending on the applications. A comparison of nozzle and build plate temperature values for the most widely used materials in the FDM method is listed in Table 2.

**Table 2:** Values of Temperature Used in the Applications of Polymeric Materials

thermoplastic materials	nozzle temperature (°C)	build-plate temperature (°C)	ref
PLA	200–210	60	(60)
ABS	225–260	80–90	
PETG/PET	225–245	85	
PP	205–220	85–100	
PC	260–280	110	

To trap the heat in the printing area of the 3D printed products for ABS filaments, the 3D printer should be closed from all sides. Because ABS filaments can be affected by temperature change easily. All filaments may emit odors during the printing process. Although PLA filaments do not emit a foul odor because of its plant-based properties, ABS filaments do emit a distinct odor.^61,62^ Thanks to the diversity in industrial applications, it has a very important opportunity to improve the properties of ABS and open new areas of application. New application areas may increase its competitiveness with other polymeric-based 3D printing materials. Moreover, there are also other thermoplastics such as PETG, PET, and PPy that can be used in 3D printing process. PETG has high strength resistance and low-cost materials, and they are utilized in many fields such as medical, automotive, aviation, building, and electrical-electronic applications.^52^ On the other hand, PET is one of the most recycled polymeric materials in high-volume commercial and consumer applications because it is widely used in plastic packaging applications as a recycling material in the beverage industry.^63^ PPy is another polymer material and it has gained popularity very quickly because of having the lowest density among commercial plastics.^64^

### 2.2 Additive Manufacturing of Polymeric Structures for Energy Conversion Applications

**Figure 2:**
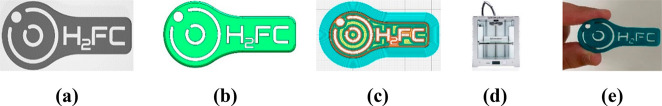
Production steps of 3D printed products with the FDM method: (a) 3D CAD model, (b) conversion STL file to designed sample, (c) slicing process, (d) 3D printing, and (e) 3D printed product.

Rapid prototyping, transforming complex structures into products, reducing printing errors, and improving mechanical properties. These are some of the main factors that may have increased the development of AM technologies. FDM and the multijet fusion (MJF) method are commonly used in 3D printing with polymer-based filaments.^65^ In the FDM method, a thermoplastic polymer filament is used in 3D printing for designed products. Due to the thermoplastic property of the polymer filament, it provides an important advantage for this method that allows fusing together during 3D printing. Then, it solidifies at room temperature after the 3D printing process is finished. Layer thickness, width, filling rate, and printing speed of the filaments are the main parameters that affect the mechanical properties for the formation of parts. Low cost, high speed, and simplicity of production steps are the main advantages of the FDM method. However, it has poor mechanical properties, poor surface quality, and a limited number of materials are their main disadvantages.^66,67^ In Figure 2, the production steps of the 3D printed model using the FDM method can be seen.

**Figure 3:**
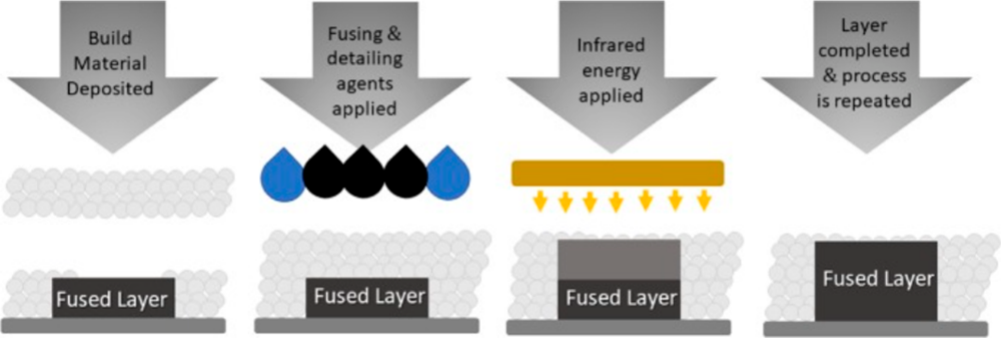
Demonstration of the MJF method involving the application of a polymer powder layer. Reprinted with permission from ref (68). Copyright 2018 Elsevier.

As seen in Figure 2, the first step of 3D printing is to create a 3D object using computer-aided design (CAD) software. The second step is to convert the 3D object to the STL (standard triangle language) file format. The third step is to separate layers of the object converted to STL format into layers with a slicing program. The fourth step is to set different printing parameters, such as the number of layers, thickness, and fill rate of the objects, and then it is sent to the 3D printer to create the product. In this method, generally polyamide 12 (PA12), polyamide 11 (PA11), and glass beaded PA12 polymer powders are used. PA12 is widely used in the multijet fusion (MJF) method. In Figure 3, the stages of the MJF method can be seen.

In this method, the production step is started by deposition of a layer of PA powder on the plate. A black ink fusing agent is applied to the powder bed and contains an infrared absorbing agent. Moreover, a substance is added to the powder bed to prevent the fusion of the particles and to enhance resolution. In this method, polymer heating is obtained as the melting agent absorbs the IR radiation and transforms it into thermal energy that allows the material to fuse by passing planar infrared rays over the powder bed to form a layer. Then, the build plate moves down to form the 3D part, and this process is repeated as a layer-by-layer production.^68^

#### 2.2.1 Conductive Polymer-Based Materials and Electrochemical Applications

PLA and ABS polymer thermoplastics’ electrical conductivity can be increased by the addition of various conductive materials. Conductive materials for 3D printing are usually obtained using metal, carbon, and polymer composites. By the addition of different conductive carbon materials (such as graphene, carbon black, nanofibers, and carbon nanotubes) in different ways, composite materials gain conductive properties.^69^ For example, a graphene-based PLA filament is produced by Black Magic 3D (BM), and it is commercially available as a “Conductive Graphene PLA Filament”. The black-colored BM PLA filament has a 0.6 Ω/cm volume resistivity value. This conductive PLA filament has mechanical strength higher than that of nonconductive PLA and ABS filaments. Thus, conductive graphene/PLA filaments are utilized in many application areas, such as sensors, printed circuits, telecommunications, medical devices, aerospace, and automotive sectors.^70^ A carbon-based PLA filament is produced by Proto-Pasta, and it is commercially available as a “Carbon Black Conductive PLA Filament”. This conductive PLA filament is used in many fields, such as low-voltage circuit applications, touch sensor areas, and touch screen pens. In addition, the Proto-Pasta PLA filament has a volume resistance of 30 Ω/cm for 3D printed parts perpendicular to the filament layers.^71^ A metal-based conductive PLA filament is produced by the Multi3D company, and it is commercially available as “Electrifi Conductive PLA Filament”. This conductive PLA filament has a brown color and a very low volume resistance of 0.006 Ω/cm. This filament is used in many fields, such as electrical circuit, electrochemical, and sensor applications.^72^ In the literature, Proto-Pasta, Black Magic, and Electrifi Conductive PLA Filaments have been used widely in 3D printing methods in electrochemical applications. For example, Vernardou et al.^73^ prepared electrodes for lithium-ion batteries using 3D printing with a graphene-based PLA filament. They fabricated the electrodes with a 3D printer, which had a dual extruder. They used a conductive PLA filament with a resistance of 0.6 Ω/cm. They also investigated the electrochemical properties of the 3D printed electrodes in a 1 M LiCl aqueous solution. They concluded that graphene-based conductive PLA filaments can be used as high-performance electrode materials.^74,75^ In recent years, the investigation of 3D printing methods in electrochemical application areas has increased.^76−79^ Electrodes for electrochemical energy conversion reactions have been obtained as 3D printed with metal and polymer-based materials. At the same time, 3D printing technology has provided a new approach to material production for a variety of applications because of their low costs. Baş et al.^80^ prepared the 3D printed anode electrodes for microbial electrolysis cells using conductive PLA filament (copper-based Electrifi filament). To increase the mass transfer inside the cell, electrodes have been designed in different geometries (rod, 1-cycled spiral, 2-cycled spiral, 3-cycled spiral, and 4-cycled spiral) and produced using the 3D printing method. They used cheese whey wastewater as an electrolyte, and a two-chamber microbial electrolysis cell with different shaped 3D printed electrodes to perform the electrochemical analyses. They interpreted that the organic content of the waste and the electrode geometry increases the microbial electrolysis performance and hydrogen production. In the literature, many reports on 3D printable polymer materials have been presented using PLA/graphene filaments,^81,82^ ABS/carbon black filaments,^83^ polypropylene/carbon black filaments,^84^ polybutylene terephthalate/carbon nanotube/graphene,^85^ and carbon nanofiber/graphite/polystyrene composite filaments.^86^ In the production of 3D printed electrodes using thermoplastic materials, carbon nanotube, graphene, and carbon black materials were mixed to increase the electrical conductivity of electrodes.^87,88^ However, electrochemical or physical deposition techniques were required to improve their conductivity to the desired level. It increases both the electrochemical activities and conductivity of the electrodes by deposition with electrochemically active nanomaterials, such as graphene and polypyrene,^89^ as well as noble metals.^90^ Moreover, commercially available PLA and ABS filaments in 3D printing technology have provided an advantage in the manufacturing of the electrodes without the need for an extrusion step. For example, Bin Hamzah et al.^91^ produced 3D printed ABS/black carbon electrodes by the FDM method and investigated their electrochemical behavior. They prepared 3D printed electrodes in both horizontal and vertical directions. When the performance of the electrodes was compared, they observed that the vertically printed 3D printed electrode showed a more advanced current than the horizontally printed electrode. Moreover, they concluded that the conductive surface areas of all 3D printed electrodes were equal in their capacitive measurements. Electrochemical activation of graphene/polymer-based filaments are also another issue for the electrochemical energy conversion studies.^92^ Thanks to the activation techniques, the amount of PLA is reduced to improve the electrode’s conductive media. João et al.^93^ studied the use of 3D printed electrodes for fuel bioethanol quality control using the FDM method. The electrodes were prepared by a mixture of carbon black and Proto-Pasta PLA filaments. The electrodes were produced in hollow cubes of 4 cm × 4 cm with a wall thickness of 2 mm. Prior to using the electrodes, they applied a polishing process to prevent possible leaks. They also performed an optimized chemical/electrochemical processing step in the electrochemical cell. Then, nonconductive polymeric material is removed from the surface of the working electrode to provide higher conductive layers. Their work concluded that the 3D printed CB/PLA electrode has exhibited a good conductivity at low currents after chemical or electrochemical surface treatment, and thus successfully completed for fuel bioethanol analysis. To improve the conductivity of PLA-based conductive filaments, electrochemical Cu coating is also a useful method.^94^ Application of Cu coating can provide an opportunity for using different shaped geometries to design electrodes, capacitors, sensors, and electrical circuits. Hüner et al.^95^ prepared electrodes by a 3D printing method using carbon/conductive PLA filament. To increase the conductivity and electrochemical performance of the electrodes, Ni–Cu binary coatings of different volume ratios were deposited electrochemically on the 3D printed electrodes. According to their results, the kinetic performance of Ni–Cu-coated 3D printed electrodes increased compared to the uncoated 3D printed electrode. Moreover, they determined that the resistance value of the Ni–Cu-coated 3D printed electrodes decreased by 99.5%. In another study, Foster et al.^81^ produced 3D printed electrodes for the oxygen evolution reaction (OER) and hydrogen evolution reaction (HER) using the FDM method. They produced graphene/PLA electrodes for HER with a commercially available conductive filament called Black Magic. They stated that the 3D printed graphene/PLA electrode exhibited low HER catalytic activity because of their poor electrical conductivity. Production of 3D printed conductive materials is currently limited in research level for the most applications. Especially, for conductive polymeric filaments, there are gaps for improving electrical and conductive properties. In another study, Hüner et al.^96^ prepared graphene-based 3D printed electrodes using the 3D printing method and then were co-deposited with different molar ratios of Ni and Pt to examine the HER features of the electrodes in the alkaline medium. In the electrochemical measurements of the prepared electrodes, they determined that the uncoated graphene-based electrode had the least HER kinetic activity in an alkaline medium. However, they stated that HER activities increased when they coated the electrodes with Ni and Pt elements. Conductive graphene-based, carbon-based, and metal-based polymeric filaments could allow production of novel 3D printed electrodes for electrochemical applications. To increase the electrical conductivity and kinetic activity of the 3D printed electrodes it is necessary to adjust the printing parameters and electrochemical coating on the electrode surface with a thin film. 3D printed electrodes also can be used for electrochemical analysis, by replacing traditional carbon electrodes. For example, Akshay Kumar et al.^97^ prepared electrodes by 3D printing and used materials with high catalytic efficiency to improve their electrochemical performance. Then, they used an easy and cost-effective dip-coating technique for the coating of the electrodes. To examine the catalytic and kinetic activities of the 3D printed electrodes for HER reactions, the electrodes were coated with different transition metals, such as WS_2_, WSe_2_, MoS_2_, and MoSe_2_. They concluded that using dip-coated 3D printed electrodes in energy conversion applications improved the surface properties. Moreover, they also stated that the surfaces of the 3D printed electrodes can be coated with various transition or noble metals, and they may be used in electrochemical applications in future electronics, sensor, and energy storage systems. Siowwoon et al.^98^ prepared 3D printed nanocarbon/PLA electrodes with MoS_2_-coated for photoassisted electrocatalytic HER using the atomic layer deposition (ALD) method and optimized the ALD process at low temperatures. The coating of MoS_2_ on the 3D printed nanocarbon electrodes is changed between 38 and 900 ALD cycles, which is performed at low deposition temperature. They explained that the prepared electrodes have higher electrocatalytic activity, reaching an overpotential of 480 mV at lower coating cycles. Moreover, they stated that the ALD deposition technique is suitable to produce complex structures with ambiguous areas, like 3D printed objects. A list of electrochemical coatings of various metals on the electrodes prepared by the 3D printing method is given in Table 3.

**Table 3:** 3D Printed Electrodes’ Electrochemical Coating Applications

3D printing method	filaments	application field	coating material	coating process	ref
FDM	graphene/PLA filament	electrode	nickel–copper	electrochemical	(78)
FDM	conductive carbon-PLA filament	electrode	nickel–copper	electrochemical	(95)
FDM	Black Magic PLA filament	electrode	gold	electrochemical	(89)
FDM	Black Magic PLA filament	electrode	nickel–platinum	electrochemical	(96)
FDM	Black Magic PLA filament	electrode	nickel–iron	electrochemical	(99)
FDM	conductive carbon-PLA filament	electrode	nickel	electrochemical	(100)
FDM	Electrifi PLA filament	electrode	copper	electrochemical	(101)
FDM	Black Magic PLA filament	graphene/PLA composite electrode	bismuth	electrochemical	(102)
FDM	graphene/PLA filament	electrode	nickel	electrochemical	(103)
FDM	Proto-Pasta PLA filament	battery/electrode	zinc–copper	electrochemical	(104)
FDM	ABS resin	composite electrode	copper	electrochemical	(105)
FDM	Black Magic PLA filament	electrode	molybdenum sulfide	electrochemical	(106)

Kim et al.^107^ produced 3D printed objects using three different commercially available thermoplastic-based conductive filaments (Electrifi, Black Magic, and Proto-Pasta). Then, they electrochemically coated the 3D printed objects with copper for 5, 15, 30, and 60 min. They investigated the electrical properties of the 3D printed objects after the copper coating process. According to their results, the 3D printed sample prepared using the Electrifi filament and coated with copper for 60 min was the best electrode. They also claimed that the copper coating reduces the electrical resistance, increases thermal stability, and current density of the electrodes. Dos Santos et al.^99^ prepared 3D printed PLA/graphene-based electrodes for OER reactions and performed a coating process on the electrode with Ni–Fe(oxy)hydroxide as an electrocatalyst. They stated that the 3D printed PLA/graphene electrode was an effective electrocatalyst against OER reaction. They concluded that a 10% contribution of Fe in the coating solution had significant kinetic activity for OER and the initial potential of OER reactions was comparable to iridium (Ir) catalysts. For the Ni-coated 3D printed electrodes, another study was conducted by Bui et al.^100^ for HER and OER performance in alkaline media. They produced 3D printed electrodes using conductive carbon PLA filament. The 3D printed electrodes were electrochemically coated with nickel (Ni) in an alkaline environment. According to their CV results, they stated that oxidation and reduction peaks occurred in the positive and negative scanning limits for OER and HER. As a graphene-based Black Magic PLA application, Iffelsberger et al.^106^ prepared electrodes by 3D printing. They deposited electrochemically MoS_*x*_ on the surfaces of the prepared electrodes and the coating of MoS_*x*_ provided an excellent electrochemical activity for HER in an acidic medium (0.5 M H_2_SO_4_). As another electrochemical energy conversion application, graphene-based conductive PLA-based 3D printed electrodes were also studied for photoelectrochemical sensors and supercapacitor applications in the form of a circular disk. 3D printed electrodes for supercapacitor applications exhibited a specific capacitance of 98.37 Fg^–1^ and it has also supplied that promising capacitance performance with stable cycling stability to 1000 charge/discharge cycles.^108^ Utilizing of conductive materials are appropriate for 3D printing may offer novel electrodes for electrochemical applications. Morphological and structural properties of electrodes used in electrochemical applications can be arranged according to printing parameters. The composition, material, and pretreatment parameters of the polymer filaments are important in the preparation process of the 3D printed electrodes. The infill ratio, the print layer thickness, and printing orientation of electrodes are prepared using the FDM method can all be changed, and so researchers may have an opportunity to explain whether these parameters can change the electrochemical properties of carbon and graphene-based electrodes. As an important issue, changing the shape and size of different electrodes with complex geometries have not yet been sufficiently investigated. Moreover, due to the constraints in producing different geometric shapes, little is known about how 3D printed novel electrodes act in electrochemical applications.^109^ 3D printed electrodes will be able to explore novel areas for electrochemical devices and it contributes to new applications where electrodes may be designed in extraordinary geometries for battery performance where traditional geometries (cylindrical, planar, button, etc.) do not perform well.

### 2.3 Metal-Based Materials

Metal-based materials have higher demand than polymeric-based conductive PLA filaments in electrochemical energy conversion applications due to their higher conductivity values. Materials, like Ti,^110^ Ti_6_Al_4_V alloy,^111,112^ Fe–Mn alloy,^113^ bronze,^114^ Al6061,^115^ Al3003,^116^ nickel,^117^ stainless steel (SS),^118−120^ and copper^121^ are used in a metal-based 3D printing method. In this method, metal powders with particle sizes ranging from 50 to 100 μm are utilized. The use of powders with small particle sizes allows the formation of homogeneous layers. When the particle size is decreased, the minimum compressible layer thickness value is reduced. Powders with large particle sizes cause uncontrollable porosity in the produced parts.^122^ Binders such as liquid glue or laser beam are used as binding agents to glue the powders into the desired structural form.^76^ During the AM process, after the solid layer is formed, the second layer of powder is spread across the previous layer in preparation for other bonding operations.^123^ In general, a lot of different materials are used in the form of small particles of ceramic, wood, acrylic, marble, and metal powders. One of the key advantages of this technology is that unbound powder particles act as a support material during the printing process. Therefore, any support material is not necessary for the printing process. Moreover, after the printing process is finished, all the remaining powder particles can be recovered effectively. Thus, metal printers will be in a good level in five years, and they may be a game changer in production industry.

#### 2.3.1 Metallic Additive Manufacturing Methods and Their Electrochemical Applications

As a good electrode production method, the metallic AM technique has generated much interest in electrochemical energy conversion studies. It is possible to use many production techniques and surface modification methods in metallic 3D printed parts. Development in the application of AM has become very popular in electrochemical applications like battery production in desired geometries, biosensors, supercapacitors, and fuel cell systems, etc. Because it is possible to bind powder particles together using high-power laser beams to fuse powder particles just below their melting point with SLS or reach their melting temperature with SLM to combine the powder particles.^124^ These laser beam coupling systems can be used in titanium, steel, aluminum, bronze, and nickel, or precious-metal-based alloys.^125−127^ It is possible to use many production techniques and surface modification methods in metallic 3D printed parts. Development in the application of AM has become very popular in electrochemical applications like battery production in desired geometries, biosensors, supercapacitors, and fuel cell systems, etc. SLS and SLM techniques are some of the most preferred metal-based 3D printings. Apart from these methods, the EBM method, which uses electron beams instead of lasers to bind metal powders, is one of the other preferred methods. This method is seen as an alternative to the SLM technique.^128,129^ The powder bed binder jetting (PBBJ) method forms metal powders using a liquid binder. In this method, the sintering or pressing method should be used to improve the mechanical properties.^23^ In the powder directed energy deposition (PDED) or direct laser metal deposition (DLMD) method, the metal powder coming to the active area is called the melting pool. Then, it is melted with a heat source focused on this point for solid object formation.^130,131^ In the electrochemical energy conversion systems, porous electrodes show high performance in industrial processes because the larger surface area can offer major advantages over electrodes due to their higher mass transfer. For example, Arenas et al.^132^ fabricated the highly porous SS structure with the M2Multilaser (Concept Laser GmbH) 3D printing device using the SLM method. It was electrochemically coated with Ni in an acidic bath solution using a rectangular channel flow cell. They concluded that the mass transport properties of the 3D printed Ni-coated SS electrode were better than typical planar and expanded metal structures. In another study, Ibrahim et al.^133^ produced SS electrodes using the SLM technique. They aimed to obtain porous electrodes with increased surface area for use in the electrochemical field. For this purpose, they tried to determine the most suitable printing parameters using A Concept Laser Mlab Cusing brand metal printer. They concluded that, by low laser power and high scanning speed, porous structures would print more appropriately. In addition, high-cost equipment and methods were used for the processing of the metallic materials. In metallic AM, objects may be produced in high precision with desired dimensions and details. In 3D printed products from the metal powders, it is seen that there is a great advantage in the desired geometries. Thanks to the AM method, it is possible to obtain electrodes with high surface area. As a result of coating the produced products using different AM methods, properties of parts such as higher strength, corrosion resistance, conductivity, and electrocatalytic activity can be enhanced for any applications. The ability to produce unique geometries in desired dimensions means that a wide range of effective systems may be achieved in electrodes for many applications. These advantageous of metallic AM undoubtedly provides the revolutionary development of electrodes used in this field. In summary, that great innovations would be possible in the use of AM method in the field of electrochemistry.

##### 2.3.1.1 Selective Laser Melting Method

**Figure 4:**
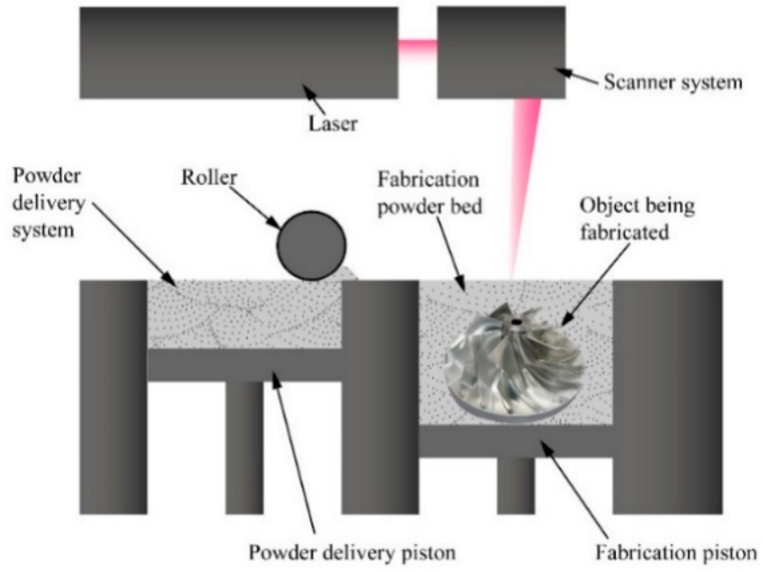
Working principle of the SLM method.

In the SLM method, the powder particles are completely melted due to the significantly high laser melting process.^134,135^ In Figure 4, the schematic illustration of the fundamental working principle of the SLM method can be seen.

**Figure 5:**
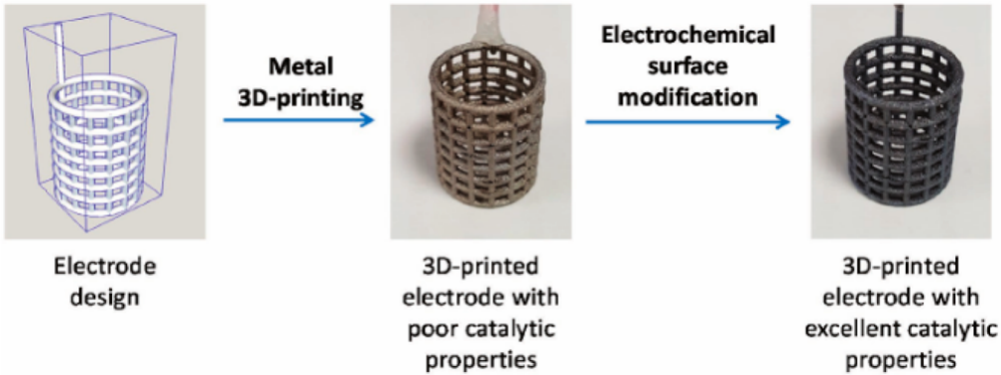
Production steps of an electrode produced by the SLM method. Reprinted with permission from ref (139). Copyright 2018 John Wiley and Sons.

This process is more suitable to create dense metal parts. In this technique, the surface roughness of the samples is higher than the other electrodes produced by the SLS technique.^136^ Moreover, the SLM 3D printed parts’ bond strength is higher than that of the SLS 3D printed parts. In general, the commercial SLM 3D printing process uses 20–50 μm particle size metal powders to print metal layers between 20 and 100 μm thickness.^137^ It is difficult to further reduce the size of the metal particles due to postpress structural defects and technical difficulties. The minimum feature size reported for SLM is in the range of 40–200 μm.^138^ As a promising energy conversion application, Ambrosi and Pumera^139^ investigated the hydrogen production performance of the SS electrode structure produced by the SLM method. They stated that the SS electrode produced by the SLM method was conductive, but it had poor catalytic properties against hydrogen and oxygen evolution reactions. To provide higher catalytic activity and corrosion resistance, Ni, Pt, and IrO_2_ were coated on SS electrode surfaces. In Figure 5, basket-shaped electrode production procedures in the SLM method can be seen.

**Figure 6:**
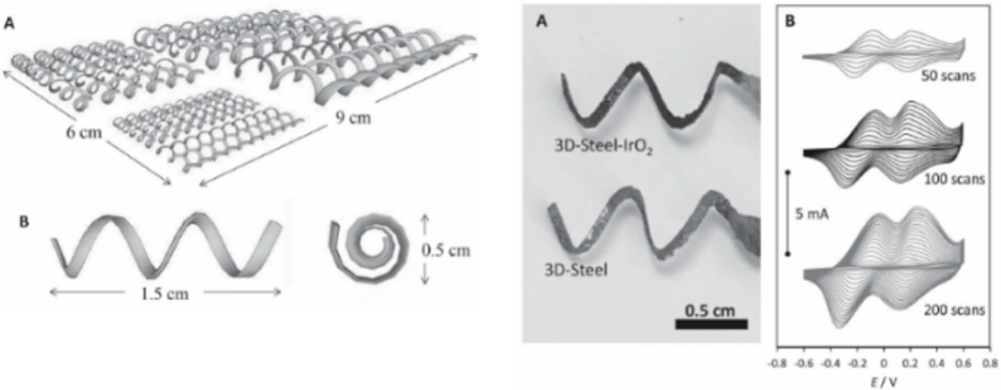
Helical SS electrodes produced by the SLM method. Reprinted with permission from ref (140). Copyright 2016 John Wiley and Sons.

As seen in Figure 5, a coated basket-shaped electrode was obtained successfully, and the direct electrolysis process may be used for similar structures. In another study, Ambrosi et al.^140^ produced SS electrodes with helical structures by the SLM method. They coated thin film IrO_2_ to increase the catalytic activity of SS electrodes. In Figure 6, helical-shaped electrodes with dimensions ranging from 1.5 to 9 cm can be seen.

**Figure 7:**
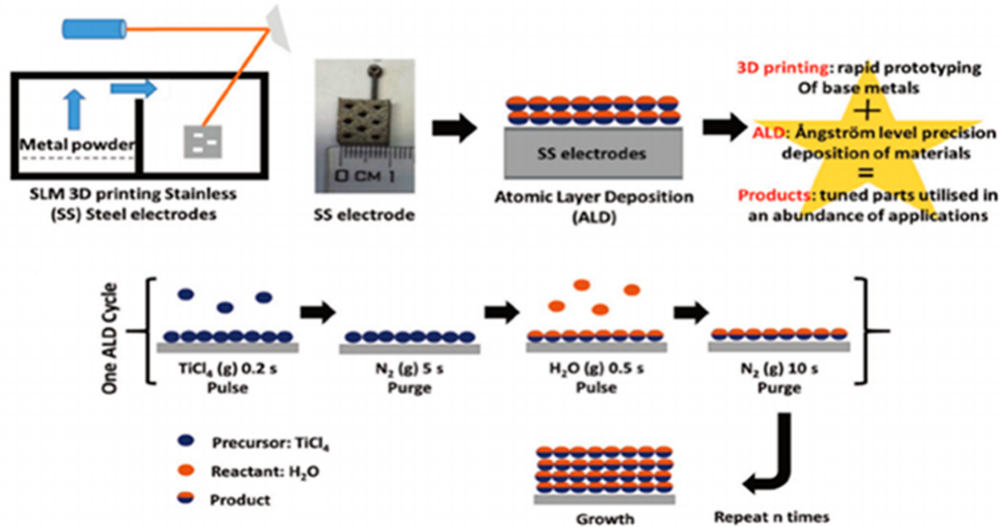
Preparation of electrodes using the SLM method and coating of the electrodes using the ALD method. Reprinted with permission from ref (141). Copyright 2019 John Wiley and Sons.

When the electrochemical performance of the IrO_2_-coated SS electrode was compared with the glassy carbon electrode. It was observed that the IrO_2_-coated SS electrode had a lower initial potential than the glassy carbon electrode. In another study, Browne et al.^141^ used ALD in combination with metal 3D printing to create active metal-based electrodes. Thus, they aimed to produce highly corrosive 3D printed electrodes without the need for any coating. While producing the SS electrodes with the SLM method, they optimized the activity by adjusting the TiO_2_ layer thickness with the ALD method. The schematic representation of the SLM and ALD methods can be seen in Figure 7.

**Figure 8:**
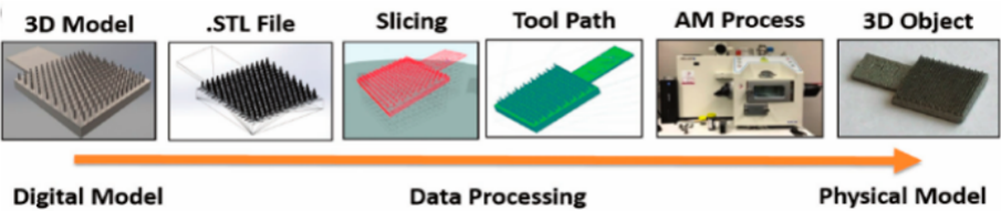
Production steps of a Ti-based conical electrode. Reprinted with permission from ref (142). Copyright 2017 John Wiley and Sons.

As a photoelectrochemistry application, Lee et al.^142^ investigated fabricating metal-based 3D printed photoelectrodes. These electrodes consisted of conical arrays, and they were produced by the SLM method. Then, their photoelectrochemical water separation performance was investigated. Due to high surface area need for efficient photoelectrochemical water separation, they prepared conical array shaped geometry. In Figure 8, the production steps of the 3D printed electrodes from Ti powder can be seen.

**Figure 9:**
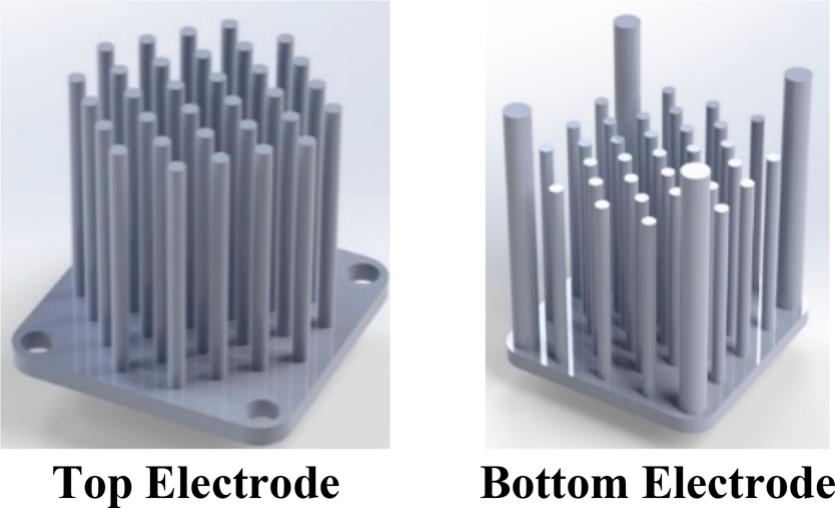
Interdigitated electrodes prepared by the SLM method. Reprinted with permission from ref (144). Copyright 2014 Elsevier.

To improve the surface area and light absorption in photoelectrochemical water separation, a conical shape was selected. They concluded that the irregularity of the conical surface structure caused by the AM process affected the electrode performance. As polymeric applications, metal-based structures were also studied comprehensively for HER and OER applications to produce pure hydrogen and oxygen in electrolysis processes. For example, Huang et al.^143^ investigated the production of electrodes with high catalytic activity for the OER reaction by the SLM method. In addition, they used the SLM method to produce a cellular SS design with high electrochemical surface area and mechanical properties and were first to do so in the literature. The SLM technique was used to optimize pore size and electrochemical surface area by comparing the 3D electrode with commercial metal foam structures. As a result of their studies, they stated that the 3D electrode produced by the SLM technique was very useful, and it might be used to produce electrodes with the SLM method, rapidly in different shapes. To obtain a staggered path for the gas flow, gas diffusion equipment may be designed to maximize the active surface area within a predefined volume.^110^ In another SLM study, Benedetti et al.^110^ designed an electrode to improve gas distribution to the active regions of a porous structure. This design is made of Ti material using Ti_6_Al_4_V metallic powder by the SLM method. After the 3D printing process, it was electrochemically coated with Pt to increase the catalytic activity of the electrode sample. According to authors’ knowledge this study has demonstrated for the first time a high surface area printed electrode with an integrated reactant delivery system. As another application for the SLM technique, Zhao et al.^144^ fabricated titanium interdigitated electrodes using the SLM method. Design of the interdigitated electrodes can be seen in Figure 9.

**Figure 10:**
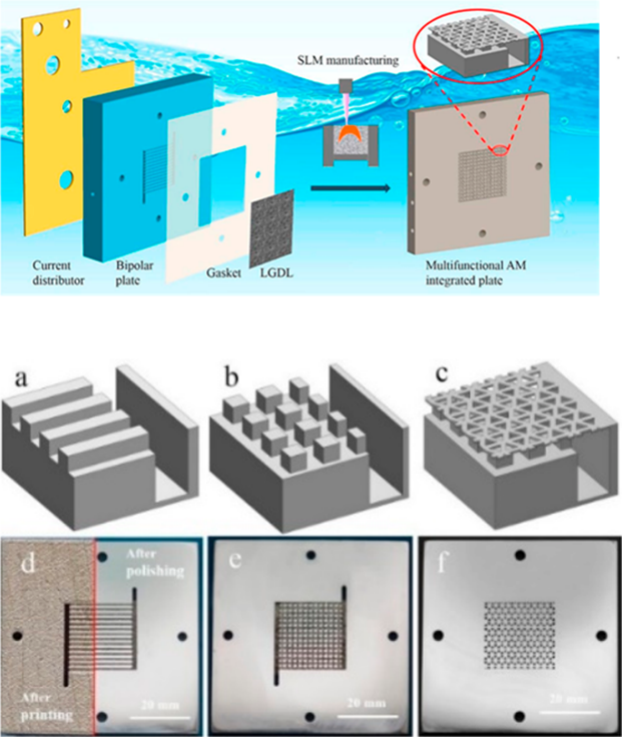
Designed and produced parts for a PEM water electrolyzers: (a) parallel flow channel, (b) pin flow channel, (c) pin flow channel with LGDL, (d) AM plate with parallel flow channel after printing and polishing, (e) AM plate with pin flow channel after polishing, and (f) AM integrated plate with pin flow channel with LGDL after polishing. Reprinted with permission from ref (147). Copyright 2018 Elsevier.

To produce this geometry, an SLM machine (Realizer SLM50) and Ti_6_Al_4_V metal powder were used for the printing process. This geometry was coated with polypyrene using the electrodeposition method, and it reached capacitance values comparable to those of the other electrodes produced by the lithography method. To obtain corrosion-resistant electrodes, the SLM method has been widely studied. For example, Kashapov et al.^145^ prepared electrodes using a 3D printer (Realizer SLM 50 model) for cleaning the surfaces of metallic products obtained with SLM technology. They used SS316 metal powder with a particle size of 20–40 μm to manufacture the electrodes. In another example, Qin et al.^146^ conducted experiments to increase the corrosion resistance of electrodes produced by the SLM method. Electrodes were fabricated by the SLM technique using Ti and Cu materials. The active surface area of the prepared electrodes was determined by a Cu wire and epoxy, and the electrochemical properties of the electrodes were investigated. According to their results, it was determined that the heat-treated samples were less likely to undergo pitting corrosion. In addition, it was stated that the waste of raw material was greatly reduced when the electrodes were printed with the SLM method by comparison of traditional methods. Yang et al.^147^ produced a current collector, bipolar plate, gasket, and gas diffusion layer parts for polymer electrolyte membrane (PEM) water electrolysis using the SLM method with a laser powder bed machine (Renishaw AM250). Produced samples can be seen in Figure 10.

**Figure 11:**
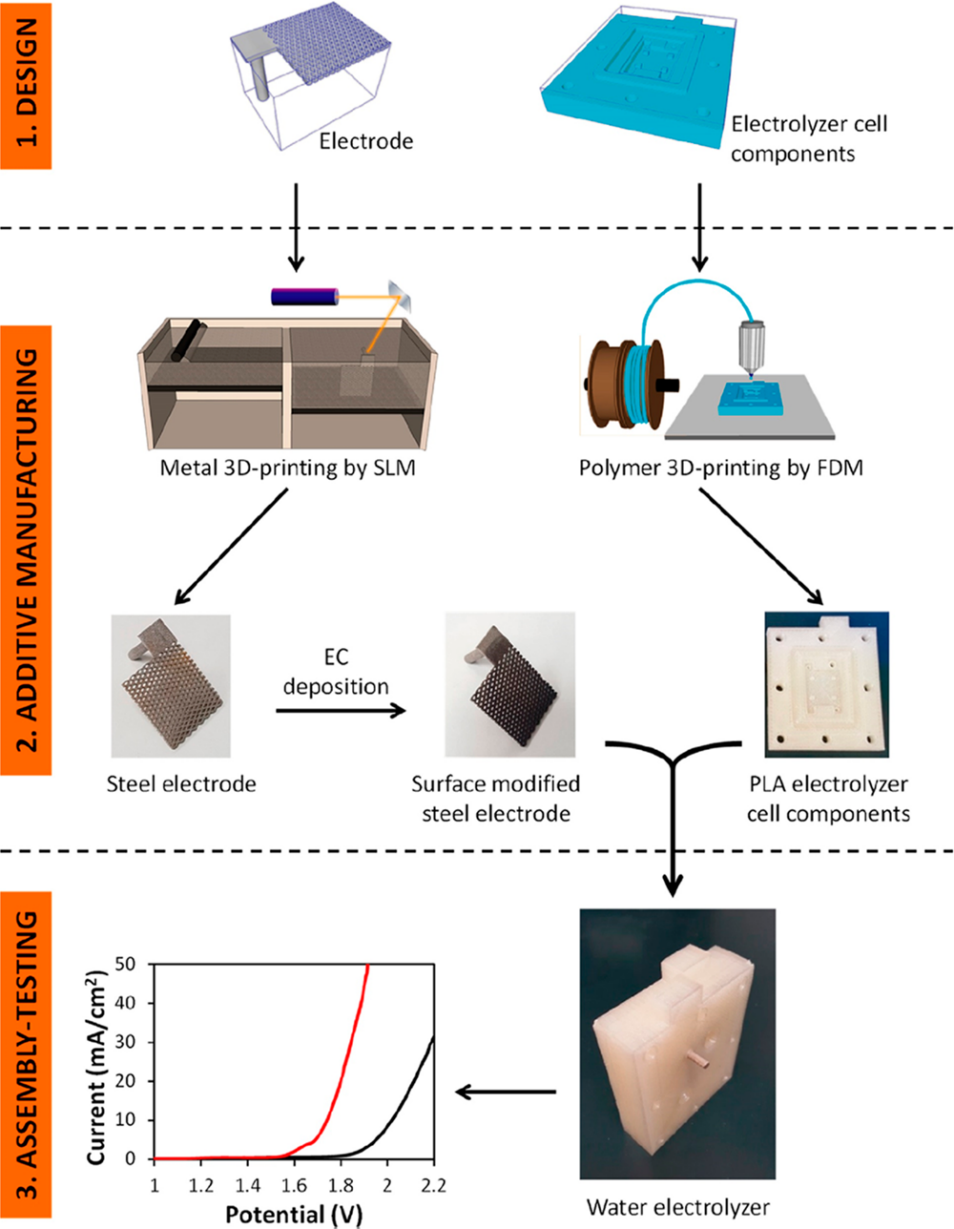
Production steps for 3D printed PEM water electrolyzer components. Reproduced from ref (148). Copyright 2018 American Chemical Society.

Figure 10a–c shows the image of the parallel flow channel, pin flow channel, and pin flow channel, respectively. The images of AM bipolar plates after polishing and cleaning can be seen in Figure 10d–f, and the surfaces of AM plates appear to be much smoother and better for assembling. The properties of the interdigitated bipolar plates were investigated by performing both ex situ and in situ experiments. At 80 °C, for in situ tests, they achieved excellent performance at 1.716 V by 2 A/cm^2^. By designing a simpler PEM water electrolyzer cell and reducing the number of the electrolyzer parts, they decreased the contact resistance, which was very important for the PEM water electrolyzers’ electrochemical performance. In another study for PEM water electrolyzers, Ambrosi et al.^148^ investigated the production of all components for a PEM water electrolyzer by the AM method. These parts were prepared using both the SLM and FDM methods. They preferred to use SS for metal parts and the FDM method with PLA filament for the other parts. Moreover, they used the electrochemical coating process to modify the electrode’s surface and electrochemical activities. These parts can be seen in Figure 11.

To increase the catalytic activity of the metallic electrodes, the anode was coated with Ni–Fe double hydroxide films and the cathode was coated with Ni–MoS_2_. In situ tests of the uncoated and coated electrodes were performed using the linear sweep voltammetry (LSV) technique.

**Figure 12:**
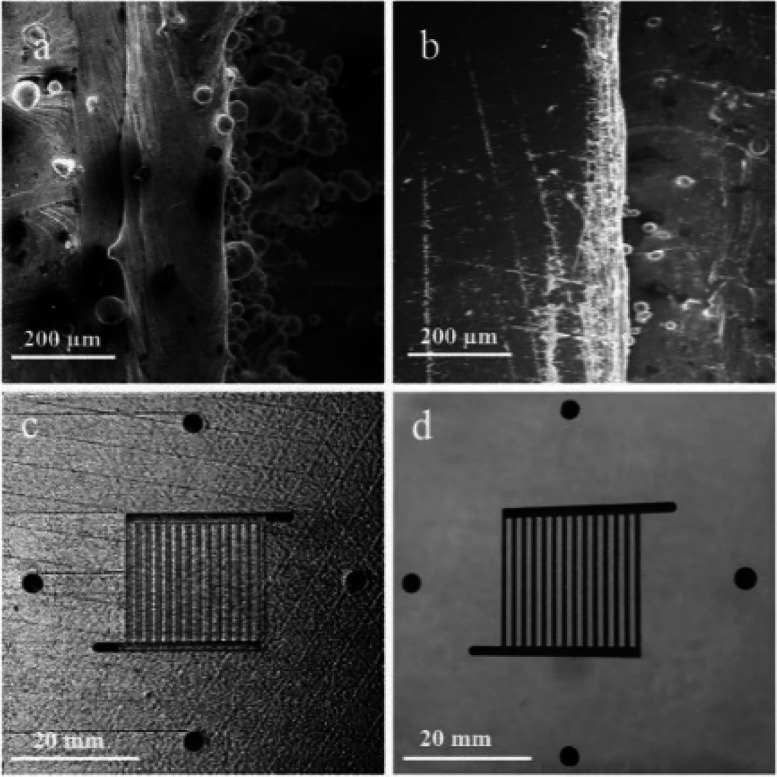
3D printed bipolar plates produced by the SLM method and SEM images of the AM cathode bipolar plate before and after polishing. (a) SEM image of land before polishing. (b) SEM image of land after polishing. (c) Image of 3D printed bipolar plate before polishing, and (d) image of 3D printed bipolar plate after polishing. Reprinted with permission from ref (149). Copyright 2017 Elsevier.

It was stated that all AM produced parts of the PEM electrolyzer cell had high electrochemical performance. In addition, Yang et al.^149^ produced bipolar plates using the SLM technique with a Magics 20A Renishaw AM250 metal printer. They concluded that the AM method may be capable of rapid and low-cost prototype development for renewable hydrogen production. Fuel cell and electrolyzer studies are very popular in metallic AM due to its flexibility to produce gas diffusion electrodes and bipolar plates. For example, scanning electron microscope (SEM) images of metallic 3D printed bipolar plates can be seen in Figure 12.

**Figure 13:**
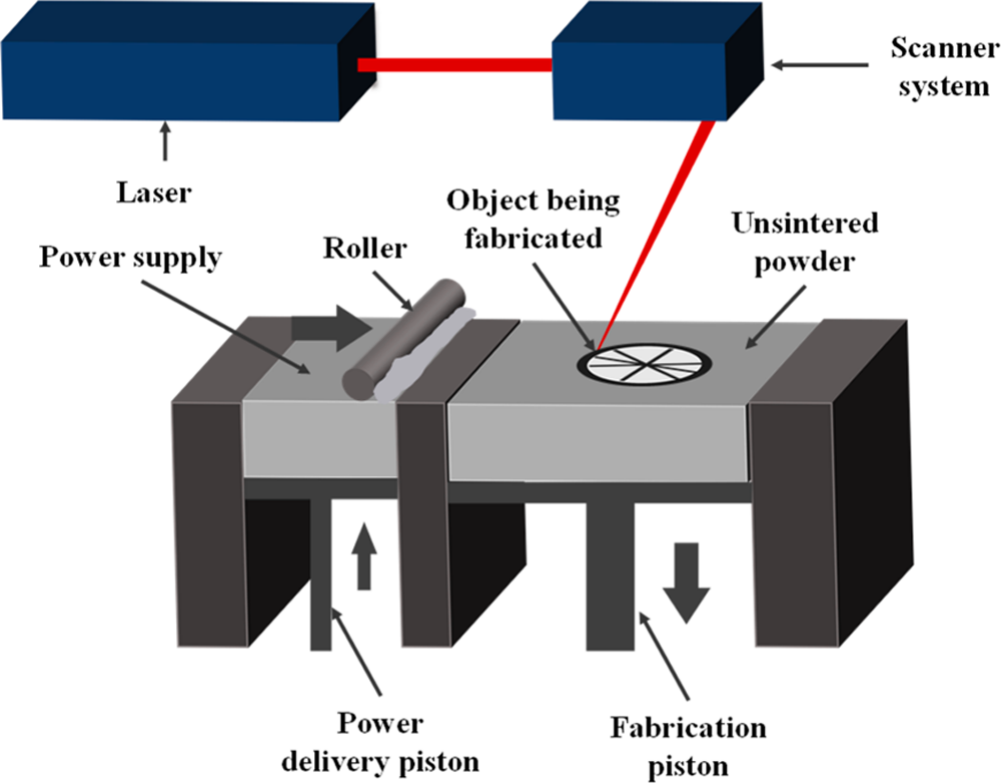
Working principle of the SLS method. Reprinted with permission from ref (155). Copyright 2014 Elsevier.

Figure 12a,b shows the SEM images of the flow channel before and after polishing, respectively. Before polishing, the surface of the bipolar plate has rough surface, and melting pool on the surface of the flow channel can be seen. Figure 13c,d shows the surface area of the 3D printed cathode bipolar plates before and after polishing, respectively. Thanks to the polishing process, the surface of the bipolar plates is become smoother and most of the excess SS powder is removed. As can be seen in Figure 12, polishing process is very important for the SLM method after 3D printing of the energy conversion device equipment’s. In another study, Laleh et al.^150^ studied the production of high relative density SS316L specimens in a jet impingement system. SS316L powders in the size of 5–40 μm were used in the SLM method. During the process, the powder bed was preheated to a temperature of 200 °C and kept in a purified argon environment until the oxygen level dropped below 100 ppm before fabrication. These parameters were chosen as the preliminary trials to produce a high-density material. The powder layers were scanned relatively in a meander scanning strategy by rotating 67° between the layers. These results indicated that the SS316L specimens produced by SLM had higher hardness and lower corrosion resistance compared to the commercially available electrodes. Moreover, in another corrosion resistant electrode study, Yang et al.^151^ improved the corrosion resistance of electrodes produced by the SLM method. In their study, they used Al-12Si metal powder to produce electrodes with two different geometries. The geometries produced by the SLM method were compared to conventional manufacturing techniques. They prepared electrodes for electrochemical measurements using copper wire and epoxy to examine the electrochemical properties of the specimens. According to electrochemical measurements and weight loss analysis, electrodes produced by the SLM method with Al-12Si metal powder showed better corrosion resistance than the as-cast Al-12Si alloy in NaCl aqueous solution. It was concluded that the difference of corrosion resistance between Al-12Si alloys produced by different methods was due to the silicon particle size in the microstructure. It was stated that the parts produced by the SLM method had better mechanical properties and worse corrosion properties than the casted parts. In addition, the production of electrodes using the SLM method was seen as among the promising methods in the field of electrochemical applications. The electrodes manufactured by the SLM technique for the electrochemical energy conversion systems are listed in Table 4.

**Table 4:** Electrochemical Applications of Electrodes Prepared by the SLM Method

powder	after process	application field	ref
SS	TiO_2_ coating	photoelectrochemistry	(141)
SS	electropolishing	OER electrode	(143)
titanium	cleaning	supercapacitor	(144)
titanium	cleaning	photoelectrochemistry	(142)
SS	MoS_2_–Ni, Ni/Fe coating	electrolyzer	(148)
SS	Pt, Ni, IrO_2_ coating	electrochemical cell	(139)
titanium	Pt coating	gas reactant transport	(110)
SS	IrO_2_ coating	electrochemical cell	(140)
titanium	annealing	rotating plasma electrode	(152)
SS	heating	plasma electrolyte	(145)
titanium–copper	heating	corrosion test cell	(146)

As seen in Table 4, SLM electrodes are generally produced with titanium and SS materials. The reason for this selection might be their high corrosion resistance and durability in alkaline and acidic environments. Several processes also possible to apply produced electrodes for performance improvements like coatings, surface treatments etc. Taking into consideration for the application areas of the electrodes, it is seen that the SLM electrodes appeal to a very wide range compared to other 3D printing methods.^153^

##### 2.3.1.2 Selective Laser Sintering Method

Another important metallic 3D printing method is the SLS method. In the SLS method, a high-energy laser beam is used for the sintering process. This laser sinters the powder material and fuses it together. The printing bed is preheated to sufficient temperature by filling it with inert gas to create a non-oxidative atmosphere.^154^ Building materials may be selected from polymer, glass, ceramic, and polymer composites. An illustration of the 3D printing method with the SLS method is given in Figure 13.

In this method, parts can be produced with a particle size of approximately 200 μm.^156^ At the same time, the SLS method is suitable for processing many different materials like 3D printing process polymer–metal powders, ceramics, polycarbonate, nylon and nylon-glass composites, and hydroxyapatite.^157^ This method is also highly preferred in the production of energy conversion materials. For example, Alayavalli et al.^158^ produced a graphite bipolar plate directly for methanol fuel cells by the SLS method and used phenolic resin as a binder. They determined that the pores of the tested parts under liquid pressure were completely closed and there was no leakage. For acidic environments, the bipolar layers should be both corrosion resistant and easily modified to any geometry. Therefore, bipolar plates are produced using graphite, non-noble, or expensive noble metals. Moreover, their compatibility with the channel design has an important place for the PEM electrolyzer. It has been stated that bipolar plates consist of 23–48% of the total cost of the PEM electrolyzer.^159^ Therefore, it is aimed to reduce the cost and material consumption with new production methods such as 3D printing. For example, Guo et al.^160^ integrated the branching structures of a tree leaf on bipolar plates. While designing the bipolar plates, they used Murray’s law to define the optimum configuration in biological circulation systems. According to both numerical and experimental studies, they reported that bioinspired interdigitated designs significantly improved fuel cell performance by 20–25% compared to traditional flow field designs. In another energy application with the SLS technique, Dobrzański et al.^161^ prepared electrodes to use in silicon solar cells. They investigated appropriate mixing ratios using different mixture combinations and they used two different silver powders with different particle sizes to fabricate the electrodes. According to their results, the silver powder could not be used in the preparation of the contact layer without SiO_2_ due to many cracks in the silicon plates. As high temperature fuel cell, solid oxide fuel cells (SOFCs) are another promising application for the SLS 3D printed electrodes.^162^ Ni electrodes may be sintered on yttria-stabilized zirconia (YSZ) material for lower contact resistance and high-performance SOFC applications by optimizing laser scanning speeds (200–6000 mm/s) and laser power (20–190 W).

##### 2.3.1.3 Direct Laser Metal Deposition Method

**Figure 14:**
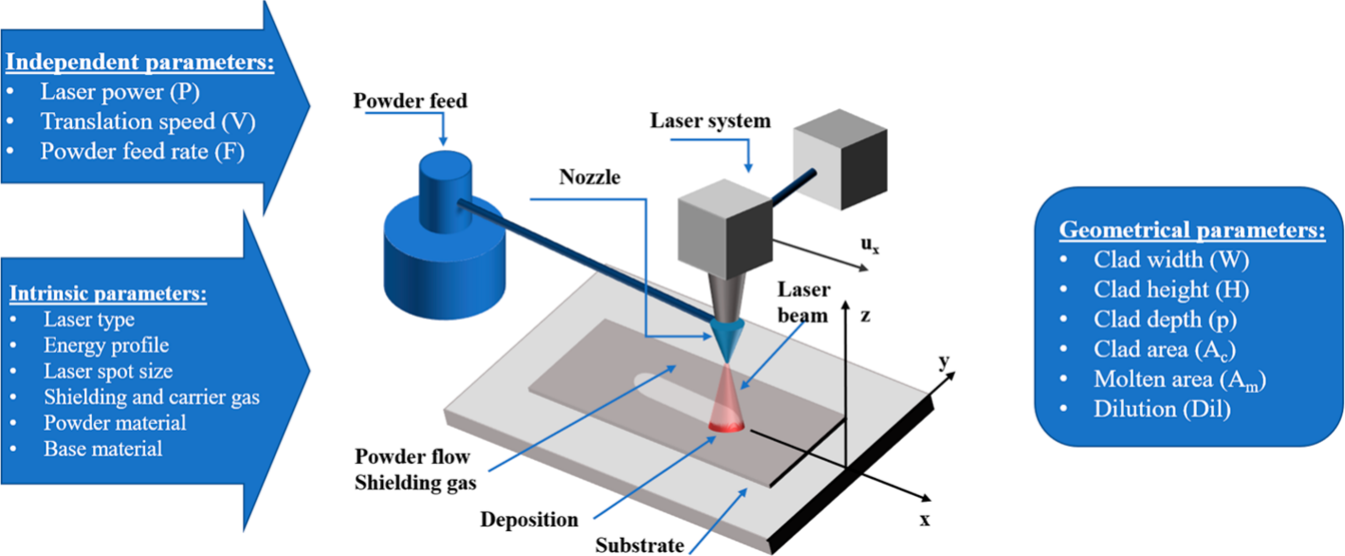
Schematic representation of the DLMD/PDED method.

Another important metallic AM method is the DLMD method. In DLMD method, or powder-directed energy deposition (PDED), account for three main parts: a 4 or 5 axis robotic arm, a powder injection feedstock, and a focused laser used as a heat source.^124^ Although the laser is commonly used, electron beam, plasma, or electric arc can be also used as heat sources.^163,164^ In Figure 14, the schematic illustration of the DLMD method can be seen.

In the DLMD/PDED method, raw powder materials are injected from the stock system and are melted by the heat source. Then, the molten material is deposited on the target surface. After the deposited material solidifies, it is bonded to the substrate layer-by-layer.^165^ This method is a highly flexible 3D printing method for the manufacturing of devices in the medical field or medium- and large-scale repairs.^166^ In this method, changing the thickness of the printed products by adjusting the power values of the heat source or the powder flow rate are the main advantages.^167^ For example, Benarji et al.^168^ investigated the corrosion behavior of electrodes produced by the PDED method. The electrodes were prepared using SS316 metallic powder with a particle size of 45–105 μm and were heat treated after sanding. It was observed that the electrodes produced by the PDED method had a lower corrosion rate than the SS316 samples produced by conventional methods. In addition, it was stated that the decrease of the ferrite phase of the SS316 electrode with the application of the heat treatment temperature caused an increase in the corrosion rate. Thus, as mentioned in this study PDED method may change the structure of the SS316 material. For the DED method, another application was conducted by Melia et al.^169^ They investigated the effects of microstructure and machining processes on the SS304L electrode 3D printed by the DED method. They used 45–90 μm powder to fabricate the electrodes and they stated that the corrosion resistance of the electrodes might be increased with a higher cooling rate. As one of the most used techniques for 3D printing metals, DED method can easily produce a heterogeneous material with desired properties with successive and simultaneous deposition of different materials. Thanks to this method, contribution to the literature can be provided with different studies by improving the product quality, shortening the manufacturing time, increasing the building volume, and material diversity. Apart from metals and their alloys, the DED method may be possible to direct ceramic processing for oxide and carbide-based ceramics or high-temperature boride or nitride-based ceramics. It is also foreseen that coatings or small-sized special cast ceramic structures may be prepared using the DED method for electrochemical energy conversion studies.

##### 2.3.1.4 Coating Applications for Metal-Based 3D Printed Electrodes

In electrochemical applications, it is necessary to improve the electrochemical properties and increase the corrosion resistance of the electrodes obtained by metal-based 3D printing methods because during the electrochemical reactions, especially in OER, highly corrosive media has contact with the electrode surface. For example, oxidation reactions occur on the anode side of PEM water electrolyzers and causes high overpotentials for the cells.^170^ To overcome this highly oxidative media, a coating process should be done by high catalytic and corrosion resistance materials. The coatings of the 3D printed electrodes prepared by metal-based powders are given in Table 5.

**Table 5:** Some Coatings for Metal-Based 3D Printed Electrodes

printing method	printing material	method	coating material	application field	ref
SLM	SS	atomic layer deposition	TiO_2_	photoelectrochemistry	(141)
SLM	SS	electrodeposition	MoS_2_–Ni and Ni/Fe double hydroxide	electrolyzers	(148)
SLM	SS	electrodeposition	Pt, Ni, and IrO_2_	electrolyzers	(139)
SLM	SS	electrodeposition	Ni	flow cell	(132)
SLM	titanium	electrodeposition	Pt	gas reactant transport	(110)
SLM	SS	electrodeposition	IrO_2_	electrochemical system	(140)
SLS	graphite	electrodeposition	Ni	DMFC electrode	(158)

According to Table 5, the SLM method is the most common method in the 3D printing process. Low raw material costs and easy application to any geometry may be the reason for the widespread use of SS. In the literature, the electrodeposition method, which is a relatively easier method compared to other methods, has been preferred for the coating process. Metals such as Ni, Pt, and Ti are selected as coating materials due to their higher catalytic activity and corrosion resistance. As given in Table 3, the application field of 3D printers and metal-based electrodes have a wide range in electrochemical energy conversion systems.

### 2.4 Other Materials and Their Electrochemical Applications

AM, which is widely known as the 3D printing technique, is used as a highly flexible technology that can be applied to conventional thermoplastics and thermosets, ceramics, carbons, epoxies, and cyanate esters, as well as a combination of other materials.^171,172^ In Table 6, comparison of the positive/negative aspects of other materials used in the 3D printing method and their applications is listed.

**Table 6:** Comparison of the Positive/Negative Aspects of Other Materials Used in the 3D Printing Method and Their Applications

materials	applications	positive aspects	negative aspects	ref
ceramics	•SOFCs and SOECs	•control of porous structures	•limited option of ceramics for 3D printing process	(173, 174)
	•automotive and aerospace industry	•easy printing of complex anatomical structures for human body organs	•extremely high melting point of ceramics	
	•biomedical	•reduction in production time	•dimensional precision errors and low surface quality	
		•providing better control over the microstructure and composition	•sintering or bonding process may be required after the 3D printing of ceramic materials	
		•no need for any molding		
epoxy-based resin, photoresin, or hydrogel	•automotive sector	•easy printing of large parts	•high cost	(175, 176)
	•chemical	•high accuracy	•poor mechanical strength	
	•health and biomedical (tissue, spine surgery, neurosurgery, and traumatology, etc.)	•very good surface quality	•fragile parts	
		•postprocessing such as sanding, and milling is not required	•low part life	
		•no need for any molding		
thermoset polymers	•automotive and aerospace sectors	•thermal stability	•brittleness	(177, 178)
	•marine	•chemical and solvent resistance	•poor impact resistance	
	•energy sector	•environmental stability	•inhomogeneous polymer architecture	
	•biomedical	•mechanical strength		
		•low cost		
		•fast production		
cyanate esters (CE)	•aerospace	•low dielectric constant	•it is not widely used in 3D printing because it is difficult to link photopolymerizable groups to their chains	(179, 180)
	•electronics	•low moisture absorption		
	•satellite communications	•high thermostability		
	•insulations and adhesives	•excellent water uptake		

Thermoplastics and thermosets come to the fore in the 3D printing process, especially because they are accessible and common materials in FDM. However, material selection for thermoplastics is mostly limited to PLA and ABS filaments. Thermoset polymers (epoxy resin, polyester, melamine, urea, etc.) is a stronger polymer compared to thermoplastics and they are more suitable to high temperature and toxic chemical environment applications because they maintain their size and shape owing to the strong covalent bonds between polymer chains.^181,182^ Ceramic or concrete materials can be produced by 3D printing methods with pores and without any cracks via optimization of parameters and adjustment of good mechanical properties. 3D printed ceramic products have occurred a trend to tailor materials with a high strength-to-weight ratio, and it is simplified the formation of complex ceramic lattices for many applications.^52^ However, compared with metals, polymers, and other materials, ceramics-based materials have one of the most critical challenges in AM method due to their extremely high melting temperature. With the increasing interest to 3D printed components of SOFCs and SOECs, studies focused on 3D printed high temperature electrochemical devices become popular due to their advantageous. Therefore, the 3D printing process has a very important place to overcome these basic limitations and reliability issues of manufacturing of SOFCs by enhancing their durability and specific power per unit volume and mass. However, the use of the 3D printing process in SOFC manufacturing is still in development stage, and researchers are displayed great efforts to bring it to a higher technology level.^183−185^ For example, Masciandaro et al.^184^ and Xing et al.^186^ have produced 3D printed YSZ electrolyte self-supports for utilization in SOFCs. They stated that the 3D printing method is a promising technique to obtain electrolyte self-support in SOFC applications. In another study, Jia et al.^187^ prepared the 3D printed YSZ electrolyte supports used in monolithic SOFC stacks with the SLM method. They stated that will have great potential for the development of SLA 3D printing processes of ceramic preparation in SOFC stacks and 3D printing technology will contribute to the future commercialization of SOFC stacks. Therefore, AM methods that can precisely utilize this kind of materials to produce fully functional, low-cost, high-efficiency energy conversion and storage devices are of great importance. It is noted that the 3D printing process has great potential in the production of electrochemical energy conversion and storage devices (electrodes, supercapacitors, etc.) compared to traditional production methods along with the use of environmentally friendly materials. Moreover, chemically active materials like catalysts are at the center of energy conversion applications. For this reason, the selection of a suitable active functional material is crucial to obtain high performance in the electrochemical reactions. Carbon-based materials such as graphene, graphene oxide (GO), carbon black (CB), carbon fiber (CF), and carbon nanotube (CNT) are often used as catalysts, supports, and electrodes in energy conversion applications.^188,189^ These materials have extraordinary mechanical, chemical, electrical, and optical properties. Therefore, carbon-based materials combined with AM technology have attracted substantial attention from the research community in energy storage and electrochemical energy conversion applications like batteries, electrodes, supercapacitors, and catalyst support.^190,191^ Moreover, carbon materials with different conductive properties can be gained conductive properties in different ways, and these materials can be quickly obtained as energy materials using different types of 3D printing methods.^83,192,193^ For example, Bian et al.^194^ produced 3D porous carbon anode electrode structures using the 3D printing method to improve power generation in microbial fuel cells (MFC). Compared with 2D flat anode materials, they stated that 3D porous carbon anode structures have a larger surface area, good mass transfer, excellent biocompatibility, and an increase in their electrochemical performance. Moreover, they commented that with the use of 3D printing technology, the pore sizes of the 3D anode electrodes can be adjusted by optimizing the surface area and mass transfers for the best MFC performances. 3D printed porous carbon materials are widely used for supercapacitors and battery electrodes. Idrees et al.^195^ proposed a 3D printed porous supercapacitor based on the use of activated carbon derived from packaging waste. They concluded that the supercapacitors made with the extrusion-based 3D printing method have a capacitance of 328.95 mFcm^–2^ at 2.5 mA. They stated that this high capacitance value is due to the porous carbon used as the active material and the high loading of activated carbon materials on the electrodes. Considering all these circumstances, these materials based on 3D printing technology and their applications will provide an opportunity for further research on 3D printable materials in electrochemical energy conversion applications in the future.

## 3 Different Geometric Shapes in the Additive Manufacturing Processes for Electrochemical Energy Conversion Applications

Contrary to popular belief, 3D printing methods offer a wide opportunity for energy materials. Different geometric shapes are obtained by combining the products produced by the 3D printing method. It is possible to obtain parts, such as electrodes and bipolar plates with 3D printing methods in the energy field. The production stages of these products and geometry structures are very interesting. These different geometric shapes have common points in terms of both production techniques and their application areas. The geometric structures produced using different methods such as SLS, SLM, FDM, SLA, DIW, and IJP should be compared. In Table 7, electrodes produced using the different 3D printing methods can be seen.

**Table 7:**
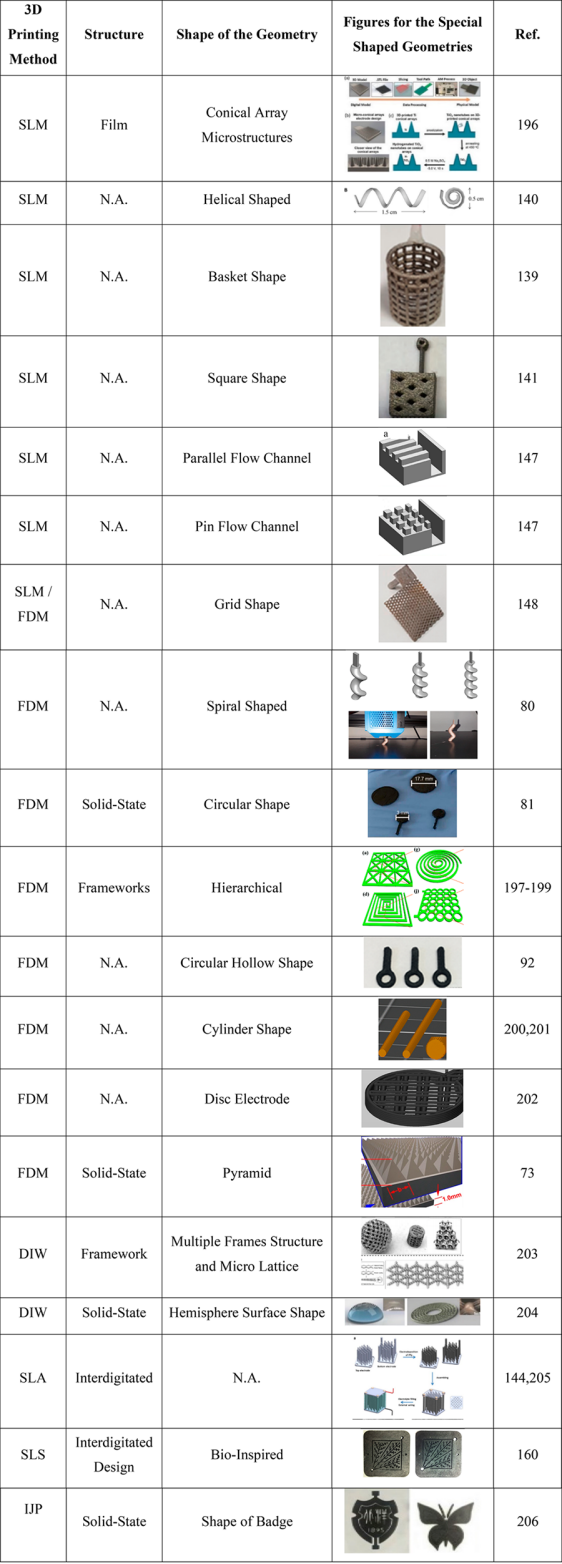
Different Shaped Electrodes Prepared by Various 3D Printing Methods^73,80,81,92,139−141,144,147,148,160,196−206^

As seen in Table 7, different geometries for several applications have many advantages in terms of their techniques. One of these advantages is the significant increase in the surface area because of their geometric shapes. Today, the geometric structures of classical electrodes, which are preferred in many applications, are insufficient to develop these systems. To determine geometric shapes used in 3D printing methods, the electrodes are named as interdigitated and framework according to their structural properties and spatial dimensions.^203^ For example, Arthur et al. stated that it would not be an appropriate approach to 3D print thick electrodes for batteries to store more energy.^207^ Geometry designs with an interdigitated structure are arranged mutually by interlacing. These are located in such a way that the anode and the cathode are positioned opposite to each other in the spatial plane.^208^ It was stated that these three-dimensionally interlocking structures minimize the ionic path length between the electrodes in a thick cell.^209^ It was also concluded that the ohmic losses decrease with the lower distance between the interdigitated and framework electrodes compared to other conventional electrodes. Long et al.^210^ examined the energy capacity and active surface area properties of electrodes in order to compare the advantages of 3D design interdigitated electrodes with 2D parallel plate electrodes. According to their results, they stated that the electrodes with conventional planar battery configurations have a much lower ohmic resistance than conventional batteries. Bowen et al.^211^ used a similar geometry structure in their study and they stated that the high voltage obtained was due to the structure of the geometry in the interdigitated electrodes. Furthermore, film-structured geometries can be 3D printed in a thin layer. The difference between these electrodes from conventional electrodes is a solid structure that can be designed in microstructures. In addition, it is possible to add polymer or fibers during the printing of 3D film electrodes. For example, since the interdigitate has a greater height than the film structure, the anode and cathode are always interdigitated in pairs in this structure. It was determined that when using the larger height interdigitate, more porosity is provided by increasing the active surface area of the electrodes.^203^ When the framework of the electrodes was examined structurally, they had a porous structure like a sieve. Thanks to this porous structure, they are frequently used in areas, such material loading. The geometric designs of the electrodes have shown unlimited variability. For example, Cheng et al.^204^ stated that the electrodes are subject to shrinkage and structural damage during the fabrication. They have performed electrodes with a self-supporting mesh hemisphere surface design to avoid degradation. In the analysis measurements, they concluded that the radial array designs with a spherical surface have a higher capacity than the conventional solid-state batteries. In this way, they stated that the 3D printed electrodes are compatible with electronic devices, and it is possible to use complex structures by the help of a 3D printing. As a result of these studies, the importance of charge transfer in electrochemical systems was emphasized, and it was stated that a continuous conductive network structure is needed for electron transfer in electrodes.^212^ Thus, AM technology provides structural integrity by improving the geometric structure designs and increasing the surface area in electrochemical energy conversion devices. The preparation of electrodes with different geometries using 3D printing methods can contribute to decreasing ohmic losses and improving their performance by increasing the amount of catalyst loaded on the electrodes. Thanks to increasing performance improvements have created the need to make compare the geometric designs of the electrodes prepared in 3D. It is especially designed for use in electrochemical energy storage devices such as supercapacitors and batteries. For example, it is determined that conical array, microstructures, helical shaped, basket shape or square shape structures provide higher power and stability than traditional 2D electrode designs. In addition, in the future studies it is expected other unique shaped designs will be prepared for electrochemical energy conversion devices by 3D printing technologies.

## 4 Conclusions and Future Perspectives

The production of complex parts or geometries, which are difficult to produce with traditional manufacturing methods, can be achieved using AM technologies without the need for any mold or production line. As an emerging technology, the AM method provides potential benefits in the electrode manufacturing sector, and it recently paved the way for the development of novel designs in industrial applications. Herein, we showed that AM technologies not only decrease the waste materials used in the manufacturing stages of products but also reduce energy consumption required during the production process. Moreover, the AM method has been accepted as one of the new generation solutions in the production novel electrodes in the fields of energy storage, energy conversion, and electrochemical applications. It is difficult to produce flow channels, such as electrodes and bipolar plates, which are utilized in energy applications with machining methods due to both the cost and complexity of the geometric structures. Therefore, the AM method has become increasingly popular in terms of freedom of design, material savings, and ease of generation of complex structures. A wide variety of materials, from polymer materials to metals, ceramics, thermosets, resins, and esters, may be 3D printed using different methods, with rapid advances in AM technologies. However, there are not many studies on the applicability of other materials in electrochemical studies using the AM method owing to still in development. Therefore, expanding the selection of materials for 3D printing of electrochemical device components, as well as research and development in electrochemical energy conversion applications, are still topics to be explored. In addition, AM enables the use of a wide variety of printable materials, which will open new opportunities in the design and application areas of 3D printing technologies. The 3D printed production of complex geometries and electrodes for electrochemical applications using the AM method will lead the way to electrochemical transformation in different geometric shapes in the future. As a very important result, these geometric shapes may be formed as wearable flexible technologies that are compatible with not only the human body, but also any animal body. Thanks to flexible biosensors, machines that interact with human learning communication may be provided by flexible structures are able to be produced by AM method. They may be also used in the development of wearable battery systems compatible with the human body or systems that can facilitate the design phase of vehicles with fuel cells. Therefore, in the future, the use of this technology will increase in various areas including R&D level and industrial applications.

## List of Abbreviations

ABS: Acrylonitrile-Styrene-Butadiene
ALD: Atomic Layer Deposition
AM: Additive Manufacturing
BM: Black Magic 3D
CAD: Computer Aided Design
CB: Carbon Black
CE: Cyanate Ester
CF: Carbon Fiber
CLIP: Continuous Liquid Interface Production
CNT: Carbon Nanotube
DLMD: Direct Laser Metal Deposition
DLP: Digital Light Processing
EBM: Electron Beam Melting
FDM: Fused Deposition Modeling
GO: Graphene Oxide
HER: Hydrogen Evolution Reaction
LMD: Laser Metal Deposition
LSV: Linear Sweep Voltammetry
LOM: Laminated Object Fabrication
MFC: Microbial Fuel Cell
MJM: Multi-Jet modeling
OER: Oxygen Evolution Reaction
PA: Polyamide
PA11: Polyamide 11
PA12: Polyamide 12
PBBJ: Powder Bed Binder Jetting
PBIH: Powder Bed and Inkjet Head 3D printing
PC: Polycarbonate
PDED: Powder Directed Energy Deposition
PE: Polyethylene
PEM: Polymer Electrolyte Membrane
PEI: Poly(ether imide)
PEEK: Polyether Ether Ketone
PET: Poly(ethylene terephthalate)
PETG: Poly(ethylene terephthalate)-Glycol
PLA: Polylactic Acid
PP: Plaster Based 3D Printing
PPy: Polypropylene
PPS: Polyphenylene Sulfide
PPSU: Polyphenylsulfone
PS: Polystyrene
PU: Polyurethane
SEM: Scanning Electron Microscope
SLA: Stereolithography
SLM: Selective Laser Melting
SLS: Selective Laser Sintering
SOECs: Solid Oxide Electrolyzer Cells
SOFCs: Solid Oxide Fuel Cells
STL: Standard Triangle Language
SS: Stainless Steel
UV: Ultraviolet
VP: Vat Polymerization
